# The Influence of Particle Shape and Surface Roughness of Fine Aggregates on the Technological Properties of Glass-Fiber-Reinforced Thin-Layer Concrete

**DOI:** 10.3390/ma19010214

**Published:** 2026-01-05

**Authors:** Ramune Zurauskiene, Asta Kičaitė, Rimvydas Moceikis

**Affiliations:** 1 Department of Building Materials and Fire Safety, Faculty of Civil Engineering, Vilnius Gediminas Technical University, LT-10223 Vilnius, Lithuania; asta.kicaite@vilniustech.lt; 2 UAB Betonika, LT-21146 Consolis, Lithuania

**Keywords:** fiberglass, fiber-reinforced thin-layer concrete, granite sifting, particle shape index, particles surface roughness, workability, segregation index, building facade panels

## Abstract

Various methods for classifying and evaluating the shape, size, and surface texture of sand particles are examined, highlighting their impact on concrete mixture properties. This study emphasizes the role of particle morphology in determining concrete workability and segregation, particularly in glass-fiber-reinforced (GRC) thin-layer concrete for building facade panels. The effects of different aggregate types on concrete workability and segregation are analyzed, showing that aggregates with spherical particles and a lower elongation index improve mixture consistency and reduce segregation. Three types of fine aggregates were used (instead of quartz sand in the mixtures, natural sand and granite screenings were chosen, which would be a sustainable alternative to quartz sand), and thin-layer glass-fiber-reinforced concrete using aggregates of different shapes was characterized by layering the mixture. The workability and segregation of fine-grained fiberglass-reinforced concrete mixtures depend on the aggregate particles’ shape. Up to 50% of quartz sand can be replaced with granite siftings or natural sand, as measured by the segregation index, as calculated according to the method proposed in this paper. Increasing the amount of natural sand from 10% to 50% also increases the segregation index from 1.9 to 2.6, and when using granite sifting aggregates, it rises from 2.6 to 3.5. Aggregates with spherical particles are more suitable for this thin-layer GRC concrete, if we examine the consistency parameters of fresh concrete and the possibilities of working with it in real production conditions.

## 1. Introduction

Fine aggregate affects the technical and economic performance of concrete, and much research has been conducted in this area, but little research has been done on the influence of aggregate shape on fine GRC. With the rapid depletion of natural pit sand resources in recent decades, fine crushed stone aggregates are increasingly preferred in the construction industry.

Mineral and rock debris mainly form due to rock weathering, and a number of classification schemes, which are being improved and harmonized, have been developed for these materials. Sediments in monodisperse rock are carefully sorted, resulting in the predomination of one granulometric fraction, while in bidisperse rock, the bulk of the particles accumulate in two granulometric fractions. Sand particles differ in origin, size, chemical and mineral composition, and shape, including predominantly round, partially round, and angled particles. The size of the particles and their other most characteristic features—shape, roundness degree, and surface roughness—are mostly determined by the physical decomposition of their rocks of origin and the distribution of the debris material formed (transfer, deposition). The shape of grains is defined by the ratio of their axes and changes over time due to the smoothing of corners and edges. Zingg [1] distinguished four main grain shape classes according to axial length ratios based on the triaxial ellipsoid: isometric, oblate, prolate, and bladed grains.

Sphericity is defined by the similarity ratio of a sphere and a given grain’s surface areas at equal volume [2], expressed as the sphericity coefficient Ks, with a spherical grain having a sphericity of 1; the less a grain resembles a sphere, the lower its sphericity value. Sphericity depends on the primary particle shape and can be determined either by formulas or visual comparison of a sample grain against benchmarks. Sphericity and roundness typically increase evenly—as one parameter increases, so does the other—and depend on particle size. The surface characteristics of fine aggregate grains are usually determined microscopically. Various names are used to describe the surfaces of particles, usually expressing the number of pits or roughness level, as well as assessing their depth, width, and the nature of various formations.

Particle shape is usually determined in a narrow range of one granulometric fraction. As the particles become larger, their degree of smoothing increases; therefore, particles in larger fractions generally have higher degrees of smoothing than those in smaller ones.

Approaches to determining the size, shape, and surface nature of rock particles can be divided into comparative, visual, geometric, and indirect methods.

Sand grain surface morphometry research methods have been used for a long time, and the general benchmark for determining sphericity and roundness is easy to use. The advantage of all these comparative methods is the possibility of quickly determining grains’ morphometric parameters; however, they suffer from the subjectivity of their determination. The previously limited capabilities of optical microscopic techniques allowed for only the general detection of grain surface roughness, angularity, or rounding. Researchers were only able to examine and describe the peculiarities of the grain surface structure as could be seen by magnifying the grain ~100 times, whereas the consequences of some chemical reactions could be seen by magnifying with an optical microscope 400 times or under >400-times magnification with other electronic devices [3].

Two- and three-dimensional research methods are used to evaluate sand particle morphometry. Some parameters are calculated based on two-dimensional (planar) parameters determined from the grain projection plane, with image analysis and visual comparison methods commonly used to evaluate the shapes of granular materials. Particle shape can be described in three different ways: by shape and sphericity (general shape), by roundness (angle sharpness), and by roughness (surface texture).

Diagrams can facilitate the evaluation of particle roundness and sphericity using regular visual aids [4]. Particles were classified by Zingg using elongation (IL) and flatness (SL) ratios [1], Krumbein provided a diagram comparing roundness [5], and Powers [6] proposed a roundness scale to visually compare and manually set roundness and sphericity values. In addition, Krumbein and Sloss proposed a diagram of the combinations of particle sphericity and roundness to evaluate their shape [7]. Cho et al. [8] modified this diagram by defining particle shape as the arithmetic mean of roundness and sphericity and adding a dotted line to the proposed shape. Researchers have also proposed a diagram to evaluate visual, two-dimensional (2D) particle angularity, with DIA and lCT analysis of the length/thickness aspect ratio (L/T ratio) as a shape parameter showing that, over almost the entire particle size range, grains have remarkably similar shape characteristics [9]. Blott and Pye combined Zingg’s studies and provided several graphs describing particle shape [10]. Deploying DIP, a camera was used to graphically visualize particles, with the results run through graphics-processing software to analyze and calculate particle shape parameters [11].

Aggregate shape and surface are important factors in determining the amount of water to be used in a concrete mix [12,13]. Quartz sand is commonly used for fine-grained concrete, and mixture consistency is good [14,15]. For the production of thin-walled products, it is difficult to apply concrete compaction agents. Therefore, an important requirement for dispersion-reinforced fine-grained finishing concrete mixes is the dispersion, compaction, and segregation resistance of the mix. Particle angularity can increase the compressive and bending strength of concrete [16] and improve adhesion between coarse particles and cement paste, which is useful in improving strength, especially bending force. Studies have shown that a fiberglass content of 1.2% has the best effect on concrete mix mobility, reaching around 70 mm [17]. Natural sand differs from most crushed aggregates (manufactured sand) in type, particle shape, and surface texture [18,19,20], with crushed aggregates having more angular surfaces. In this case (decreasing values of slump and slump flow), more water is required for concrete production [9,21]. Replacing 100% of natural sand with manufactured sand increases water demand [22]. Manufactured sand (MS) has a higher roundness value and length–area ratio, and these parameters’ distributions are larger in MS than in river sand [18]. In the case of self-compacting concrete, mobility and workability are easily compromised by the phenomena of segregation and gravity [23]. The decrease in concrete workability is affected by the type, amount, and geometry of the used fiberglass, as well as the initial mix composition [24,25,26]. In addition to the factors mentioned above, concrete workability is also affected by the length, length-to-diameter ratio (l/d), and shape configuration of the fiberglass used [27], with different amounts and lengths of fiberglass being shown to have different effects on mobility [28]. Study results have shown that the optimum fiberglass contents for workability, stability, and mechanical properties are about 1% and 4% for 12 mm and 6 mm long particles, respectively [29]. It can also be seen that the addition of short fibers (3 mm, 6 mm) results in a higher slump than with long fibers (12 mm, 20 mm). The reason for this may be the uneven distribution of long fibers throughout the concrete matrix, thereby reducing the slump of the concrete [28]. Incorporating fiberglass increases the viscosity of concrete, thus reducing its fluidity [30,31], and poor fiber dispersion significantly reduces the workability and stability of the matrix [32]. Scientists have proposed a model that has proven to be an effective tool for designing fiber-reinforced concrete mixes with selected fresh-state properties using different fiber ratios and types, but they suggest that the model should be further refined in order to account for different aggregates [33]. However, there is not enough literature on the effect of different aggregates on the workability and segregation of concrete mixes for this refining to be performed.

Since there is currently very little material on the effect of aggregate morphology on the technological properties of fiberglass-reinforced concrete, this work analyzes the effect of different aggregates (natural sand and granite siftings) on the workability (spread and slump) of concrete mixes by replacing part of the mixes’ quartz sand with natural sand and granite siftings. Quartz sand is a sought-after and expensive aggregate that can be used to make many different construction products, so reducing its amount for concrete production should be welcomed. In this study, it was important to examine particle shape in a consistent manner and identify the key index affecting segregation.

The research aimed to investigate whether granite siftings—waste produced when crushing granite rocks—are suitable for use in the production of fine-grained glass-fiber-reinforced thin-layer concrete, to what extent these aggregates can replace quartz sand, and whether natural sand is a suitable replacement.

## 2. Materials and Methods

**Figure 1:**
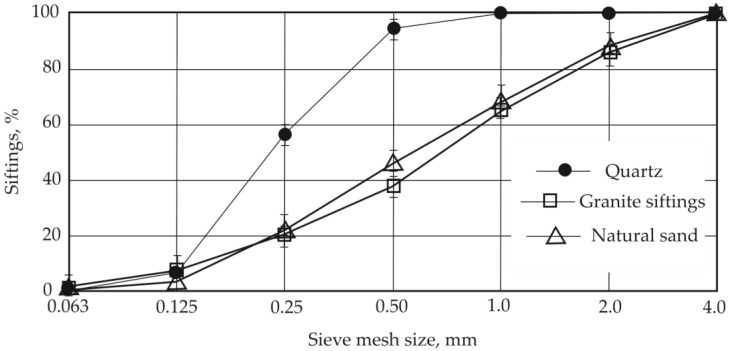
Granulometric curves of aggregates.

Three types of fine aggregates were used in the study, the first being quartz sand, a common aggregate for mixing mixtures intended for dispersion-reinforced fine-grained finishing concrete. A certain proportion of the quartz sand was replaced with crushed granite siftings in some mixtures, and with natural sand in others. The main physical and chemical properties of the fine aggregates are presented in Table 1, and their granulometric compositions are shown in Figure 1. In the production of thin-layer panels for facades, quartz sand is used, with a typical particle size of <1 mm. As this is a fairly expensive resource, this study investigated the potential of replacing it with conventional, less expensive materials. Specifically, we explored the use of crushed granite siftings (particle size < 2 mm) and natural construction sand supplied from a quarry with a usual particle size of <2 mm.

Portland cement CEM I 52.5R was used in the work, and its chemical and mineralogical compositions, as required by the standard LST EN 197-1: 2011 [34], are presented in Table 2 and Table 3.

A highly effective and widely used polycarboxylate ester-based superplasticizer was used to plasticize the cement matrix. The dry particle content is 30%, and the recommended dosage range for normal concrete is 0.8–2% of the cement mass.

Main characteristics of glass fiber: tensile strength 1400 MPa, deformation modulus 74 GPa, thermal resistance –50 °C to +350 °C, melting temperature 1100 °C, specific density 2500 kg/m^3^, fiber length 12 mm, fiber content in fiber 200 pcs, single fiber diameter, 18 μm.

In the production of our concrete mixtures, 853 kg of cement was used per 1 m^3^ of mixture. The total amount of fine aggregate per 1 m^3^ of the mixture was also 853 kg. The amount of quartz sand included varied from 50% to 100%, being partially replaced with granite siftings in GS mixtures and natural sand in NS mixtures in 10% intervals (10%, 20%, 30%, 40%, and 50%). Mixtures with granite siftings were marked GS10, GS20, GS30, GS40, and GS50, while the natural sand mixtures were named NS10, NS20, NS30, NS40, and NS50, and the fully quartz sand sample was denoted as QS. A high-performance superplasticizer, widely used to plasticize ester-based polycarboxylates in cement matrices, was added, equal to 1.1% of the cement mass; the W/C (water/cement) ratio was 0.36; and glass fibers were 2.9% of the dry matter content (Table 4).

**Figure 2:**
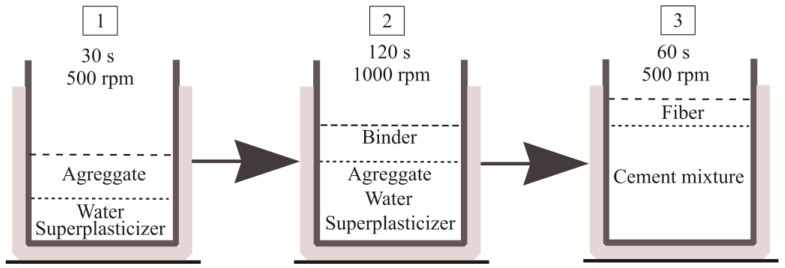
Mixture preparation scheme.

After creating a series of experimental mixes, an optimal mix preparation method was selected based on recommendations in the literature. Water, superplasticizer, and aggregate are added first, and the mix is stirred slowly for 20–30 s to completely wet the aggregate particles. The binder is then dosed, and the mix is stirred for 2 min at a maximum mixer speed of 1000 rpm until a steady consistency is reached. Fiberglass is then added and stirred for 1 min at half the maximum speed of the mixer—500 rpm (Figure 2). It should be noted that this mode is suitable for mixes where the fiberglass is evenly distributed throughout the volume and does not break down into individual fibers. The high amount of fine-grained additives in the mix makes conventional normal concrete mixing techniques inadequate, as there is not enough energy to mix the large amount of fine grains and obtain the required mix consistency [36]. For these fine-grained mixtures, the intensive mixing method was used exclusively, with the concrete mixer set up with a single high-speed (up to 1500 rpm) rotating axis and a spiral nozzle.

The cylindrical spread method is suitable for fine-grained cementitious composites with fibers up to 20 mm in length and was chosen to study the workability of our concrete mixes. A ø = 57 (*h* = 55 mm) metal cylinder was used for the test, placed on a smooth surface (glass or marine plywood) with a concrete mix poured into it. After 15–20 s the cylinder is removed, the mix is allowed to spread, and the diameter (cm) of the spread mix is measured after 10–15 s (LST EN 1170-1 [37]). The tests with each mixture were performed three times.

**Figure 3:**
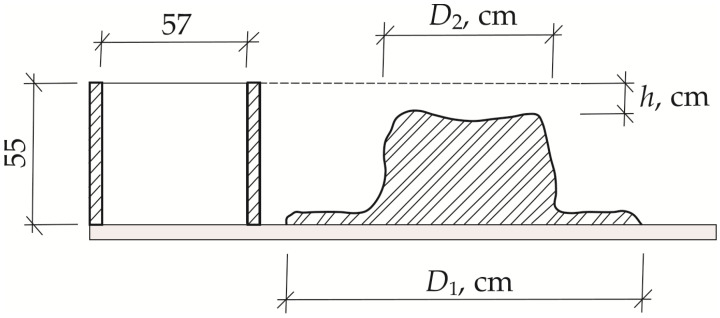
Additional parameters for evaluation of GRC mix workability [35].

Based on the diagram shown in Figure 3, we can create a parameter defining mix workability—the segregation index *W*. This is directly proportional to *D*_1_ and *D*_2_ but inversely proportional to *h*, and is expressed according to the following formula (dimensionless size):

(1) $$W\;=\;\frac{D_{1}{-D}_{2}}{h},$$

where *D*_1_ is the cementitious mix spread, calculated as the average of two measurements made in the perpendicular direction, cm; *D*_2_ is spread of the fiber-reinforced cementitious matrix, calculated as the average of two measurements made in the perpendicular direction, cm; and *h* is the mix slump (Suttard’s viscometer height), cm.

In order to better understand the effect of aggregate particle shape on thin-layer GRC concrete mix workability, the average particle shape parameter elongation index *I* was calculated:

(2) $$I\;=\;\frac{d_{1}}{d_{2}},$$

where *d*_1_ is the longer side of the aggregate particle, mm, and *d*_2_ is the shorter side of the aggregate particle, mm.

To determine particle shape, all three fine aggregates were separated into fractions by sieving through standard sieves—2–4 mm, 1–2 mm, 0.5–1 mm, 0.25–0.5 mm, 0.125–0.25 mm, and 0.063–0.125 mm—and microscopic photographs of each fraction were taken. An MIN10 optical microscope (LOMO, Saint Petersburg, Soviet Union) was used, which can magnify an image up to 104 times, with illumination from above, resolution 1.1 μm. After loading the images into AutoCAD 2020, elongation index (*I*) was determined with the help of measuring tools by selecting 30 particles from each fraction and calculating the average index value. Since the elongation index *I* is calculated as the ratio of the particle dimensions in the perpendicular direction, the scale of the images in the AutoCAD model space does not affect results.

When calculating particle shape and surface roughness index J, another parameter is introduced—surface roughness (*R*). This can be determined using the shape (elongation) (*I*) and surface roughness (*R*) parameters of the aggregate particles:

(3) $$J\;=\;I\cdot 1.9(1+\frac{R}{100})\;,$$

where *I* is the aggregate particle elongation index and *R* is the surface roughness.

The results surface roughness index obtained are based on data surface roughness presented in literature sources [4,11,38,39] and the results of elongation conducted research.

## 3. Results and Discussion

**Figure 4:**
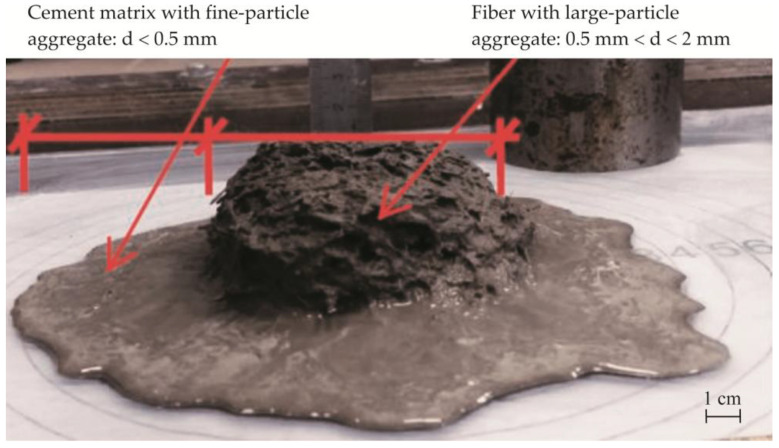
Segregation of dispersion-reinforced fine-grained concrete mix [35].

Quartz sand is the most studied and most commonly used aggregate in fine-grained concrete systems due to the particularly good mix workability achieved using this aggregate [14,40], though other materials such as silica flour and silica fume can also be used [41]. Several studies have investigated replacing the expensive quartz sand normally used in glass fiber concrete with more economical, locally available natural sand [42]. In GRC concrete, fibers tend to tangle and form clusters in the center of the flow [32], and in the fluid concrete mixes studied in this work, the ability to spread quickly and evenly and to fill the formwork of the formed product is especially important. In the production of thin-walled products, it is difficult to apply concrete compaction measures, making mix spread, slump, and segregation resistance important requirements for dispersion-reinforced fine-grained finishing concrete mixes. Mixture segregation is defined in the scientific literature as the separation of the fibers and cement matrix, as shown in Figure 4.

**Figure 5:**
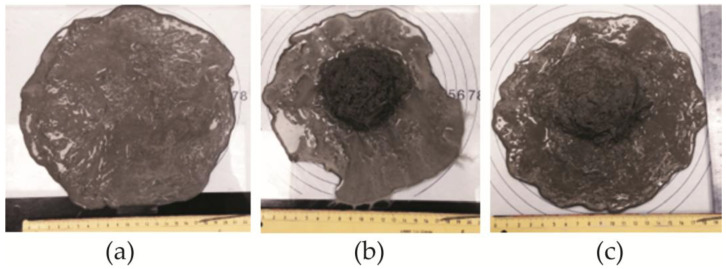
Photos of workability tests: (**a**) reference quartz matrix (100% quartz filler); (**b**) 50% of quartz replaced with granite, GS50 [35]; (**c**) 50% of quartz replaced with natural sand, NS50 [35].

In order to reduce the use of quartz sand, we investigated the workability of several different thin-bed GRC mixtures. All our samples were tested, and the most representative trends were selected for visual representation in the Results section. The use of alternative aggregates significantly decreased mix workability, in some cases resulting in high fiber–matrix segregation. The determination of regular slump according to Suttard’s viscometer height (*h*) alone was not sufficient to describe the workability of such mixes in detail, and the spread diameters *D*_1_ and *D*_2_, which characterize the segregation effect (Figure 4), must also be measured. Figure 5 shows the different spread tendencies of our concrete mixes. As shown in Figure 5a, the best spread was achieved in the mix containing only one type of aggregate (quartz sand), allowing a self-leveling GRC mix to be obtained. The spread is 22 cm and the analyzed sample reaches full slump, which is not reached when half of the quartz sand is replaced with granite screenings or natural sand (Figure 5b,c).

**Figure 6:**
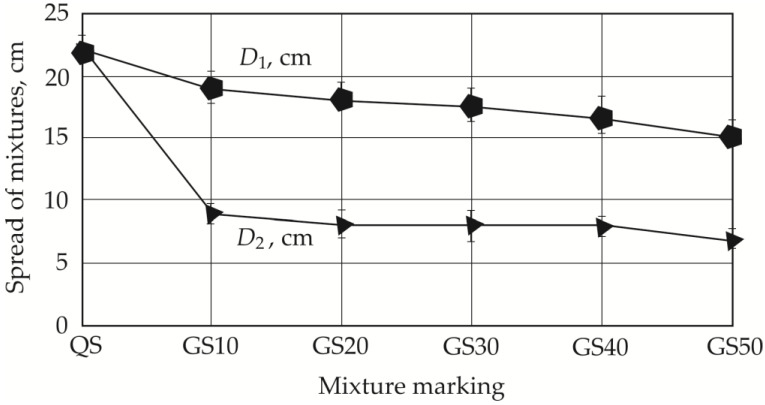
Dependency of mix spread on the amount of granite particles.

**Figure 7:**
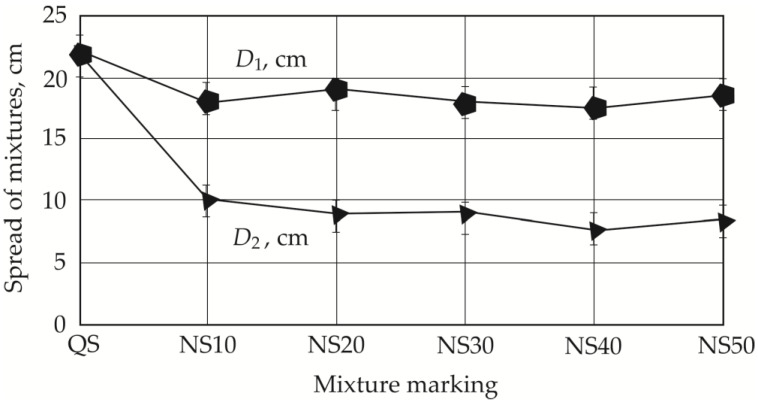
Dependency of mix spread on the amount of natural sand.

Spread can be seen in Figure 6 and Figure 7. Replacing 50% of quartz sand with granite siftings or natural sand shows a decrease in spread, with a greater spread reduction observed when using fine granite grains (Figure 6): spread *d*_1_ decreases by 31.8% with 50% fine granite grains (GS50) and 15.9% with 50% natural sand (NS50). In the case of spread *d*_2_, the same tendencies are observed when using both 10% granite siftings (GS10) and 10% natural sand (NS10) (Figure 5 and Figure 6). In both cases, a sudden decrease in the mix’s spread is observed, which can be explained by studying the elongation of the aggregate. In the first case, it decreases to 9 cm, and in the second case to 10 cm.

**Figure 8:**
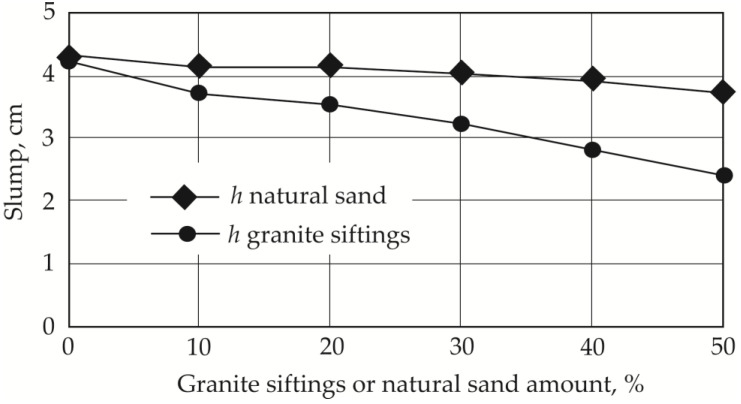
Dependency of mix slump on amount of granite siftings or natural sand.

Slump tests (Figure 8) showed a decrease from 4.3 cm to 2.4 cm (44.18%) when adding granite siftings and to 3.7 cm with natural sand (13.95%). A number of studies on regular concrete have shown that aggregate particle shape and surface characteristics are important factors in determining a concrete mix’s water–cement ratio—the larger the particle surface area, the greater the amount of water required to obtain a concrete mix with the required workability [14]. Angled particles can increase concrete’s compressive strength; however, the workability of the mix worsens [38].

**Figure 9:**
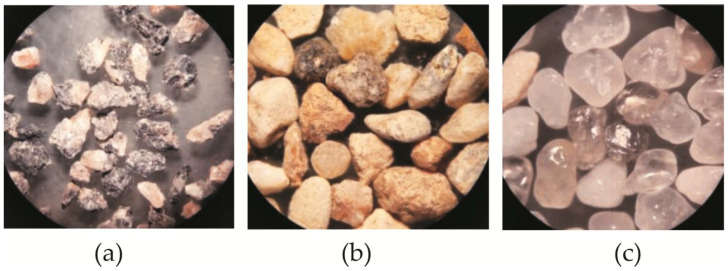
Aggregate shape analysis under an optical microscope for 1 mm particles (25× magnification): (**a**) granite siftings; (**b**) natural sand [35]; (**c**) quartz sand.

The quartz sand particles used in these studies have a spherical shape with smooth surfaces, resulting in less friction between the cement matrix, aggregate, and fibers (Figure 9c). However, more uniform and spherical sand particles, forming fewer concrete voids, will improve flow [42]. Sands with higher roundness values also have higher length–width ratios [39]. There are almost no studies on the properties of granite siftings, but analyses of natural and artificial sand surfaces have been performed. On the one hand, natural sand has a smooth and round surface due to water washing, movement, and wear over a long period of time, while manufactured sand particles have rough surfaces, sharp edges and corners, and low roundness, with more inter-particle friction and interlocking, and therefore require more cement paste to pack and lubricate [12]. Granite sifting particles have a plate-like shape with sharp edges, which increases the internal friction in the matrix and means that fibers become trapped between the larger particles while fine particles pass through the gaps together with the cement mortar (Figure 9a). Natural sand aggregate particles are shaped like irregular spatial polygons; therefore, the workability parameters of natural sand mixes are closer in composition to mixes containing the granite aggregate (Figure 9b).

Concrete consistency and segregation depend on the shape of aggregate particles, as shown by consistency and segregation studies. A number of particle shape parameters have been developed by researchers and are used in the numerical modeling of particle mix density [10]. However, other researchers have proposed using simplified shape indices (spherical mass, flatness, and elongation) to study these concrete mix consistency problems [4]. In this work, the elongation index, which can be determined by simple visual means, such as microscopic photographs or plotting software, is chosen as such a parameter. An increase in aggregate particle elongation index of just 3% reduces mixture dispersion by 10% when natural sand is used instead of quartz sand. When an irregularly shaped aggregate (granite siftings) is used, the particle elongation index increases (Table 5) by 33% compared to quartz sand, and the spread of the mixture decreases, accordingly, to 50%. Increasing the content of natural sand and granite siftings, respectively, from 10% to 50% increases the segregation index from 1.9 to 2.6 and 2.6 to 3.5. These results indicate that mixtures with a predominance of spherical particles, ensuring uniform cement matrix and fiber dispersion, are more in-demand when a well-laid pavement mixture is required.

According to the amount of each fraction left behind on different sieves, the average elongation indices for the aggregates were calculated: *I*_q_ = 1.40 (quartz sand), *I*_ns_ = 1.44 (natural sand), and *I*_gs_ = 1.87 (granite siftings) (see Table 5). The predominant fraction in quartz sand is from 0.125 to 0.25 mm, while in natural sand, the various fractions are distributed more evenly. In the case of granite siftings, the largest fraction of particles was between 0.5 and 1 mm long. According to the test results, concrete mix workability worsens with an increase in both particle elongation index and the proportion of larger particles. The highest elongation index values were determined in the case of granite siftings, as shown in Figure 8. In this case, the elongation value increases by 33.58%, and the concrete mix spread decreases accordingly to 50%. In the case of natural sand, the increase in the elongation index is only 2.86%, but even in this case, the spread of the mix decreases by 10%.

Research on conventional concretes has shown that the shape and surface characteristics of aggregate particles are important factors in determining the water/cement ratio of a concrete mix. The larger the surface area of the particles, the greater the amount of water needed to obtain a mix of the required consistency with a given amount of aggregate [38]. Angular particles can increase the concrete’s compressive strength but degrade the consistency of the mix [38]. The quartz sand particles used in these studies are characterized by their spherical shape and smooth surfaces, resulting in less friction between the cementitious matrix, aggregate, and fibers. Granite sifting particles have a plate-like shape with sharp edges, which increases the matrix’s internal friction, trapping the fibers between the coarser particles and allowing the fine-grained particles to pass through the gaps with the cementitious mortar. The regular sand aggregate particles are irregularly shaped spatial polygons, meaning that the consistency parameters of the NS mixtures are closer to those of the GS compositions.

**Figure 10:**
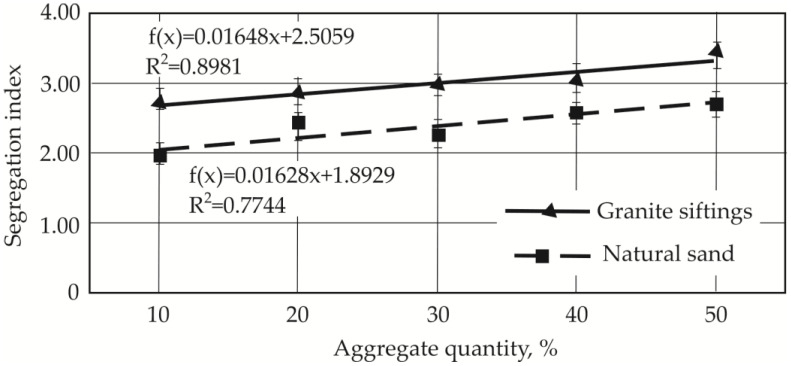
Segregation indices for compositions with different quantities of aggregates: granite sifting and natural sand [35].

**Figure 11:**
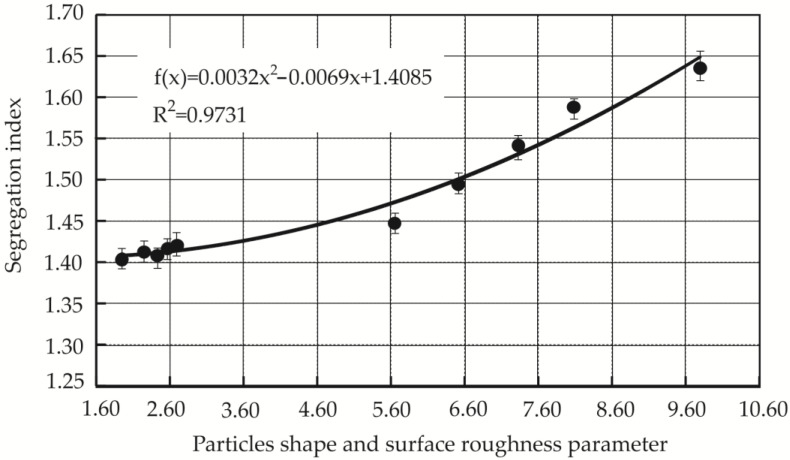
Dependence of the segregation index (*W*) on the shape (elongation) and surface roughness parameter (*J*) of aggregate particles.

Segregation index tendencies are presented in Figure 10 and Figure 11. The segregation index is a measure used to quantify the degree of separation between different components in a concrete mix. Its linear dependencies are shown in Figure 10, with our results showing that as the proportion of natural sand and granite siftings in the mix increases, so does the segregation index. This indicates a direct relationship between the included amount of these alternative aggregates and the mix’s tendency to segregate. The correlation coefficient R for the relationship between granite screenings is 0.948, while the linear correlation coefficient R for the relationship between natural sands is 0.88. The segregation index values are consistently higher for concrete mixes incorporating granite siftings than those with natural sand. At the maximum replacement rate of quartz sand with granite siftings, the segregation index is 1.27 times higher with the granite aggregate than with natural sand. The fillers differ in their particle surfaces, with sand being smooth while granite is rough.

The segregation index is influenced not only by the aggregate shape but also by its surface roughness. The particle shape and surface roughness parameter *J* was calculated using data provided from the study of fine aggregate particle surface parameters [39]. The dependence of mixture segregation index on the shape (elongation) and surface roughness of the filler particles is shown in Figure 11. This figure shows that the segregation index’s dependence on these parameters can be described by a square function, indicating that small changes in elongation and roughness can lead to larger changes in the segregation index. The dependence of segregation indices on particle shape and surface roughness parameters is strong—its correlation coefficient R is 0.986. This suggests a non-linear relationship, where changes in particle shape and roughness can significantly impact segregation tendencies in a complex manner. As the surface roughness increases, the surface irregularities become larger, the aggregates become more elongated, and the segregation index increases.

In summary, we emphasize the crucial role of aggregate characteristics in determining concrete workability and stability. Our research reveals that both the shape and surface texture of aggregates significantly influence concrete’s segregation tendencies, with rougher and more elongated particles leading to higher segregation index values. This comprehensive understanding can inform the selection and proportioning of aggregates in concrete mix designs to achieve desired workability and minimize segregation.

## 4. Conclusions

When studying the influence of fine-grained particle shape and surface roughness on the technological properties of glass-fiber-reinforced thin-layer concrete, the following main trends were identified and conclusions drawn:

1. When different aggregates are used, fiberglass-dispersion-reinforced fine-grained concrete is characterized by layering of the mixture, leading to the separation of the fiberglass and fine aggregates from the cement matrix. Layering depends on the aggregate’s particle shape. As the aggregate particle elongation index increases from 1.4 to 1.64, the segregation index of the concrete mix varies from 1.9 to 3.5.
2. The highest concrete mixture flow diameter was obtained with quartz sand and a particle elongation index equal to 1.4. An acceptable value for the segregation index of the mix can be achieved when replacing up to 50% of the quartz sand with granite siftings or natural sand.
3. The workability and segregation of a fine-grained fiberglass-reinforced concrete mix depend on aggregate particle shape, described by the elongation index and expressed as the ratio of the longer side of the particle to the shorter side. As the elongation index increases, workability worsens and segregation increases. An increase in the aggregate elongation index of 3% reduces the spread of the mixture by 10% when natural sand is used instead of quartz sand. When an irregularly shaped aggregate (granite siftings) is used, the particle elongation index increases by 33% compared to quartz sand and the spread of the mixture decreases to 50%, accordingly. Increasing the amount of substitution from 10% to 50% further increases the segregation index from 1.9 to 2.6 and 2.6 to 3.5, respectively, for natural sand and granite siftings.
4. An even spread of cement matrix and fiber is ensured for fine-grained fiberglass-reinforced concrete mixes when aggregates with spherical particles (i.e., an elongation index of no more than 1.4 based on acceptable segregation levels and technological parameters) are used. The segregation index is also important for these mixtures and needs to be assessed during technological operations. The index can be calculated according to the method proposed in this paper.
5. Aggregates with spherical particles that ensure the even spread of the cement matrix and fiber are more suitable for GRC concrete. The shape and surface roughness of aggregate particles have a decisive influence on concrete mixture segregation. This dependence is described by a square function.
6. In addition to increasing the angularity of the aggregate particles, an increase in the surface roughness of the aggregates significantly increases the segregation index of a mix.
7. The possibility of using granite siftings or natural sand instead of quartz sand has been investigated. These aggregates have different physical surface characteristics that significantly affect the consistency of concrete mixtures. Therefore, in the future, when using fine aggregates with different surface parameters, different methods of mixture compaction must be applied, taking into account the possibility of segregation.

## Figures and Tables

**Table 1:** The main physical and chemical characteristics of the fine aggregates.

Aggregate	Main Characteristics					
	CaCO_3_ + MgCO_3_, %	Al_2_O_3_, %	SiO_2_, %	Specific Density, kg/m^3^	Bulk Density, kg/m^3^	*d*_max,_ mm
**Natural** **sand < 2**	5.16	<3.0	90–93	2600	1550	12.9
**Granite** **siftings < 2**	0	14.4	70–75	2750	1530	2
**Quartz sand** **<1.25**	0	<0.6	>98.5	2650	1640	1.25

**Table 2:** Chemical composition of Portland cement CEM I 52.5R, % [35].

CaO	SiO_2_	Al_2_O_3_	Fe_2_O_3_	MgO	SO_3_	K_2_O	Na_2_O	Cr
63.99	19.84	5.24	2.99	1.55	3.05	0.78	0.76	0.062

**Table 3:** Mineral composition of Portland cement CEM I 52.5R, % [35].

C_3_S	C_2_S	C_3_A	C_4_AF
53.56	17.23	1.43	10.4

**Table 4:** GRC mixture compositions with granite and regular sand for workability investigation.

	Changed Content of Quartz Sand, %				
	10	20	30	40	50
Cement CEM I 52.5R, kg/m^3^	853	853	853	853	853
Quartz sand, kg/m^3^	767	682	597	512	427
Granite siftings or regular sand kg/m^3^	86	171	256	341	427
Superplasticizer, 1.1% by weight of binder, kg/m^3^	9.38	9.38	9.38	9.38	9.38
W/C (water/cement ratio)	0.36	0.36	0.36	0.36	0.36
Glass fibers, 2.9% by weight of solids, kg/m^3^	49.5	49.5	49.5	49.5	49.5

**Table 5:** Determination of particle shape index.

Fraction, mm	Quartz Sand		Natural Sand		Granite Siftings	
	Quantity, %	Elongation Index Iq	Quantity, %	Elongation Index Ins	Quantity, %	Elongation Index Igs
2–4	-	-	11.8	1.38	13.9	2.00
1–2	0.3	1.21	20.0	1.31	20.7	2.20
0.5–1	5.2	1.37	22.3	1.32	27.6	1.76
0.25–0.5	37.6	1.34	23.3	1.69	17.0	1.59
0.125–0.25	49.5	1.42	19.0	1.45	12.9	1.97
0.063–0.125	7.4	1.56	3.6	1.4	8.0	1.61
Total aggregate	100	1.40	100	1.44	100	1.87

## Data Availability

The original contributions presented in this study are included in the article. Further inquiries can be directed to the corresponding author.
